# Engineering mouse chymotrypsin B1 for improved trypsinogen degradation

**DOI:** 10.1038/s41598-025-94299-1

**Published:** 2025-03-25

**Authors:** Nataly C. Morales Granda, András Szabó, Zsombor Köller, Gábor Pál, Miklós Sahin-Tóth

**Affiliations:** 1 Department of Surgery, University of California Los Angeles, Los Angeles, CA 90095 USA; 2 Department of Biochemistry and Molecular Biology, Faculty of Medicine, University of Debrecen, Egyetem Tér 1, 4032 Debrecen, Hungary; 3 Doctoral School of Molecular, Cell and Immune Biology, University of Debrecen, Egyetem Tér 1, 4032 Debrecen, Hungary; 4 Department of Biochemistry, ELTE Eötvös Loránd University, Pázmány Péter Sétány 1/C, 1117 Budapest, Hungary; 5 MacDonald Research Laboratories, 675 Charles E Young Drive South, Rm 2220, Los Angeles, CA 90095 USA

**Keywords:** Chronic pancreatitis, Chymotrypsin, Trypsinogen, Serine protease, Proteases, Translational research

## Abstract

The digestive protease chymotrypsin (CTR) protects the pancreas against harmful trypsin activity by promoting degradation of trypsinogen. Recently, we demonstrated that Arg236 is responsible for the higher proteolytic activity and better trypsinogen degrading capability of human CTRB2 compared to CTRB1. Introduction of Arg236 into CTRB1, which normally carries Asp236, dramatically increased degradation of human anionic trypsinogen. Here, we explored whether we could improve the activity of mouse CTRB1 by changing Gly236 to Arg (G236R mutant) and/or by widening the substrate binding pocket (A244G mutant). We found that mutant G236R cleaved mouse anionic (T8) trypsinogen at Phe150 with 32-fold improved efficiency. In contrast, mutant G236R digested mouse cationic (T7) trypsinogen and bovine beta-casein at the same rate as wild-type mouse CTRB1. Mutation A244G reduced the activity of mouse CTRB1 against the two trypsinogen isoforms and casein. Double-mutant G236R-A244G cleaved mouse anionic (T8) trypsinogen 9.8-fold better than wild-type CTRB1 but 3.3-fold slower than single mutant G236R. Mutant G236R-A244G digested mouse cationic (T7) trypsinogen at the same rate as single-mutant A244G but degraded casein 2.3-fold slower. Taken together, the observations indicate that in the context of mouse CTRB1 the Arg236 residue increases protease activity in a substrate-specific manner, while Gly244 has an overall negative impact. The results will inform the design of preclinical mouse models with higher trypsinogen degradation ability and enhanced resilience against pancreatitis.

The digestive serine protease chymotrypsin protects the pancreas against pancreatitis by reducing intrapancreatic trypsin activity^1^. This mechanism begins to work only after the first line of defense against premature trypsinogen activation provided by the trypsin inhibitor SPINK1 is depleted and emerging trypsin activity converts the proenzyme chymotrypsinogen to its active form. Chymotrypsin, in turn, halts further increase in trypsin activity through degradation of trypsinogen, the precursor to trypsin^1–3^. The pancreas synthesizes and secretes chymotrypsin precursors in multiple isoforms; in humans the predominant forms are chymotrypsin B1 and B2 (CTRB1 and CTRB2), while chymotrypsin C (CTRC) and chymotrypsin-like protease (CTRL) are less abundant isoforms. In the mouse pancreas, CTRB1, CTRC, and CTRL are produced. Interestingly, the commonly used laboratory mouse strains C57BL/6J and C57BL/6N are naturally deficient in CTRC due to a single-nucleotide deletion in the gene^4^. In C57BL/6N mice, CTRB1 represents about 90% of the total chymotrypsinogen, while CTRL constitutes 10%^5–7^. Accordingly, in laboratory mouse models, CTRB1 mediates protection against pancreatitis through cleavage of mouse anionic (T8 and T9) and cationic (T7) trypsinogen while CTRL plays a minor role^5–7^.

In humans, CTRC is responsible primarily for the anti-trypsin defenses while CTRB2 contributes to a small degree and CTRB1 and CTRL are less important^2,8–14^. Loss-of-function *CTRC* variants increase the risk for chronic pancreatitis by 2.6–6.5-fold in heterozygous carriers^11^. The common *CTRC* variant c.180C>T (p.Gly60 =) increases risk by 1.9-fold and 5.3-fold in the heterozygous and homozygous state, respectively^12^. Furthermore, mutations in the *PRSS1* gene that render human cationic trypsinogen resistant to degradation by CTRC cause hereditary pancreatitis^3^. The protective role of CTRB2 was discovered by a GWAS study that showed that an inversion at the *CTRB1-CTRB2* locus decreased risk to chronic pancreatitis by 1.36-fold^10^. The duplicated *CTRB1* and *CTRB2* genes are found in opposing orientation with respect to each other and code for chymotrypsinogens that share 98% sequence identity. The inversion exchanges the 5’ region, exon 1 and intron 1 between the isoforms, without affecting exons 2–7, which code for the mature chymotrypsinogens. Owing to the inversion, expression of CTRB2 relative to CTRB1 increases, resulting in more efficient degradation of human anionic trypsinogen, which is susceptible to digestion by CTRB2 but not CTRB1^10^.

An important difference in the amino-acid sequence of human CTRB1 and CTRB2 is position 244 (position 226 in conventional biochemical and crystallographic numbering), which helps to shape the substrate binding pocket and thereby modifies substrate specificity (Fig. 1)^15^. Human CTRB1 has a wider binding pocket due to Gly244 and, therefore, cleaves more robustly after Trp than CTRB2 that contains Ala244. CTRB2, however, cleaves after Phe, Tyr, and Leu with better catalytic efficiency than CTRB1^16^. Another conspicuous divergence between human CTRB1 and CTRB2 is position 236 (position 218 in biochemical numbering), which contains amino acids with opposing charges; Asp236 in CTRB1 and Arg236 in CTRB2. Interestingly, other mammalian chymotrypsins tend to harbor a neutral residue at this position, typically Ser (e.g. bovine, goat, sheep, pig) or Gly (mouse, rat, cat, dog) (Fig. 1). Recently, we demonstrated that the evolutionary selection of Arg236 in human CTRB2, versus Asp236 in CTRB1, imparted CTRB2 with higher activity^17^. This explains why the increased levels of CTRB2 in carriers of the *CTRB1-CTRB2* inversion allele result in more efficient degradation of human anionic trypsinogen and protection against chronic pancreatitis. Remarkably, we found that the introduction of Arg236 into human CTRB1 dramatically (37.7-fold) increased degradation of human anionic trypsinogen; the D236R CTRB1 mutant was even more effective than CTRB2 by 7.1-fold. To extend these observations to mouse studies in the future, here we explored whether we could improve the activity of mouse CTRB1 by changing Gly236 to Arg and/or by widening the substrate binding-pocket through mutation of Ala244 to Gly. In the experiments, the enzymatic activity of single and double mouse CTRB1 mutants were tested against peptide substrates, bovine β-casein, and mouse cationic and anionic trypsinogens.

**Fig. 1:**
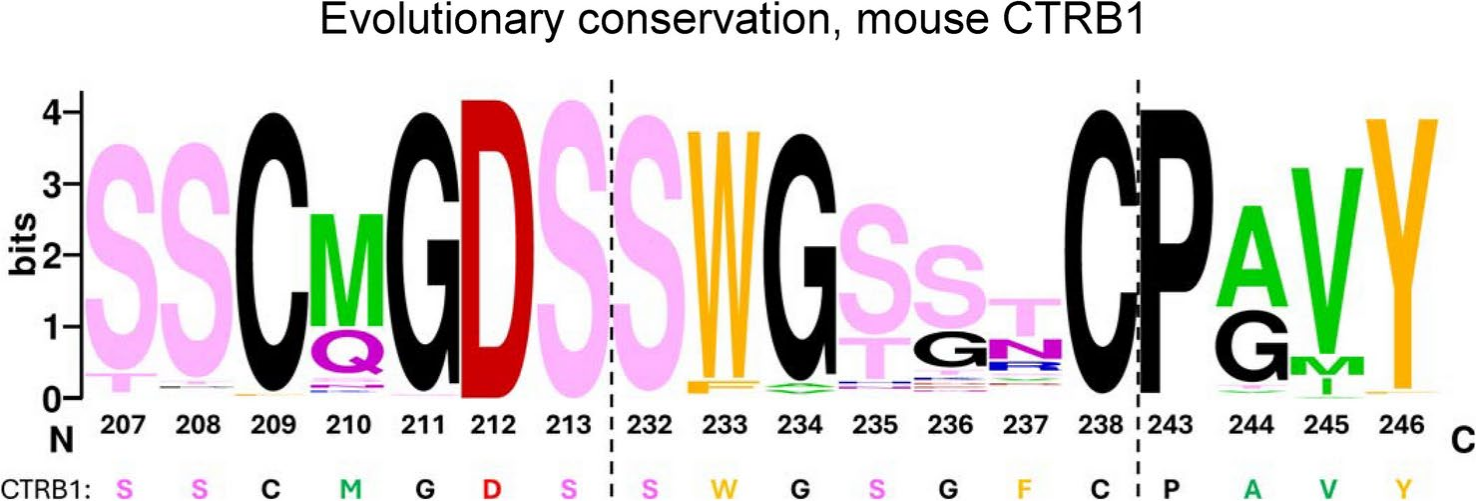
Sequence logo representation of positional residue conservation in the substrate binding pocket region of mouse CTRB1 homologues. The three sequence blocks correspond to the peptide segments that form the walls of the substrate binding S1 pocket. Amino acids are indicated with their one-letter code. The logo was created with the WebLogo application (https://weblogo.berkeley.edu/logo.cgi). The input sequence set was generated by multiple sequence alignment of natural mouse CTRB1 homologs, as described in “Materials & methods”. The height of each column indicates the level of evolutionary conservation (frequency) at the given position. Chemical characteristics of the amino-acid residues are color-coded as follows: aliphatic is green, aromatic is orange, acidic is red, basic is blue, polar without charge is pink, and Cys, Gly, Pro are black. The corresponding amino-acid sequence of mouse CTRB1 is also shown. Note that none of the homologues contained Phe237 which was present only in mouse CTRB1.

## Materials & methods

### Nomenclature and accession numbers

Amino-acid residues were numbered starting with the initiator methionine of the primary translation product. The UniProtKB entry for the mouse CTRB1 protein is Q9CR35. Historically, the two major chymotrypsin isoforms isolated from bovine pancreas were named chymotrypsin A (CTRA) and B^15^. The main determinant of their respective substrate specificity was mapped to position 244 where CTRA contains Gly244 and CTRB Ala244 (Fig. 1)^15^. The corresponding human isoforms were named CTRB1 and CTRB2 where CTRB1 is the A-type (with Gly244) and CTRB2 is the B-type (with Ala244) chymotrypsin. The mouse pancreas expresses a single isoform, named CTRB1, which is a B-type enzyme with Ala244. Thus, mouse CTRB1 is the functional ortholog of human CTRB2. The UniProtKB entries for mouse anionic (T8) and cationic (T7) trypsinogens are Q9R0T7 and Q9CPN9, respectively.

### Expression plasmids

Construction of the pTrapT7-T8 and pTrapT7-T7 bacterial expression plasmids containing the coding sequence for mouse anionic (T8) and cationic (T7) trypsinogen, respectively, were described previously^18^. Mutations F150S and D156S in mouse anionic (T8) trypsinogen were generated by overlap extension PCR mutagenesis and the PCR products were cloned into the pTrapT7-T8 plasmid using the NcoI and SalI restrictions sites. The pcDNA3.1(-) mouse CTRB1 10His plasmid harboring the coding sequence for the mouse chymotrypsinogen B1 precursor with a C-terminal 10His tag was constructed previously^6^. Mutations were introduced by overlap-extension PCR mutagenesis and the PCR products were cloned into the pcDNA3.1(-) vector with the XhoI and BamHI restriction sites. To prevent autolysis of chymotrypsin preparations, all mouse constructs carried the Y164A mutation. The expression plasmids for human CTRB1, CTRB2, and the D236R CTRB1 mutant were described recently^17^.

### Expression and purification of proteases

Mouse trypsinogens were expressed in *E. coli* and purified by ecotin affinity chromatography as described previously^18,19^. Concentration of anionic (T8) and cationic (T7) trypsinogen preparations were estimated from their UV absorbance at 280 nm, using the extinction coefficients 34,670 M^−1^ cm^−1^ and 39,140 M^−1^ cm^−1^, respectively^18^. Wild-type and mutant mouse CTRB1 proenzymes were expressed in HEK 293 T cells with transient transfection and purified from the conditioned medium by nickel-affinity chromatography according to our published protocol^20^. Chymotrypsinogens were dialyzed against 15 mM Na-HEPES (pH 8.0), 100 mM NaCl, and activated with trypsin beads (catalog number 20230, Thermo Fisher Scientific, Waltham, MA). The beads were removed by centrifugation and active chymotrypsin concentrations were determined by titration with the high-affinity pan-specific protease inhibitor, ecotin^21^. Human CTRB1, CTRB2 and the D236R CTRB1 mutant were purified previously, as reported^17^.

### Active site titration

Concentration of active mouse CTRB1 and its mutant forms was determined by titration with ecotin. A two-fold serial dilution of 100 nM ecotin was prepared in 50 µL assay buffer (0.1 M Tris–HCl (pH 8.0), 1 mM CaCl_2_, and 0.05% Tween 20), and 50 µL chymotrypsin diluted with assay buffer to 20 nM nominal concentration (estimated from the UV absorbance of the chymotrypsinogen preparation, using the extinction coefficient 47,605 M^−1^ cm^−1^) was added to each well. The 100 µL mixture was incubated at 22 °C for 30 min and the residual chymotrypsin activity was measured with 100 µL of 200 µM Suc-Ala-Ala-Pro-Phe-*p*-nitroanilide substrate (dissolved in assay buffer). Protease activity was plotted as a function of the ecotin concentration, and the chymotrypsin concentration was determined by extrapolating the linear portion of the inhibition curve to the *x* intercept.

### Enzyme kinetic analysis

Measurements were carried out in 0.1 M Tris–HCl (pH 8.0), 1 mM CaCl_2_, and 0.05% Tween 20 at 22 °C. The active chymotrypsin concentration in the assay was 2 nM. The concentration of the Suc-Ala-Ala-Pro-Phe-*p*-nitroanilide and H-Ala-Ala-Pro-Phe-*p*-nitroanilide substrates were varied in the 1.2—148 µM and 5.2–660 µM range, respectively. Rates of substrate hydrolysis were plotted as a function of the substrate concentration, and the data points (mean ± SD, n = 3) were fitted with the Michaelis–Menten hyperbolic equation to obtain the *K*_M_ and *k*_cat_ values. The Suc-Ala-Ala-Pro-Phe-*p*-nitroanilide substrate was obtained from Bachem Americas (Torrance, CA, catalog number L-1400.0250), while the H-Ala-Ala-Pro-Phe-*p*-nitroanilide substrate was custom-synthesized, as reported earlier^17^.

### Digestion of casein

Bovine β-casein (catalog number C6905, MilliporeSigma) was digested at 0.2 mg/mL concentration in 0.1 M Tris–HCl (pH 8.0) and 1 mM CaCl_2_ using 5 nM chymotrypsin, at 37 °C. At the indicated times, 100 µL aliquots were withdrawn and the reactions were terminated by precipitation with trichloroacetic acid (10% final concentration). The samples were analyzed by 15% SDS-PAGE followed by Coomassie Blue staining and densitometric quantitation.

### Trypsinogen digestion assays

Mouse anionic (T8) and cationic (T7) trypsinogens were incubated at 2 µM concentration with 1 nM or 100 nM chymotrypsin, as indicated, in 0.1 M Tris–HCl (pH 8.0), at 37 °C. To prevent autoactivation of trypsinogens, the digestion mixtures contained 80 nM human SPINK1 trypsin inhibitor (final concentration). At given times, 100 µL aliquots were withdrawn and the reactions were terminated by adding trichloroacetic acid to 10% final concentration. The protein precipitate was recovered by centrifugation and samples were analyzed by SDS-PAGE, Coomassie Blue staining, and densitometry.

### Gel electrophoresis and densitometry

Protein samples were precipitated with 10% trichloroacetic acid (final concentration). The precipitate was collected by centrifugation (10 min, 13,200 rpm, 4 °C), and dissolved in 25 µL 2 × Laemmli Sample Buffer (catalog number 1610737, Bio-Rad, Hercules, CA) supplemented with 100 mM dithiothreitol and 150 mM NaOH to neutralize the leftover trichloroacetic acid. The samples were heat-denatured at 95 °C for 5 min, electrophoresed on 15% SDS–polyacrylamide gels, and stained with Brilliant Blue R-250 (Coomassie Blue). Densitometric quantitation of the undigested casein and trypsinogen band intensities was carried out with the ImageJ software (version 1.52a). Full, uncropped gels are shown in the Supplementary file.

### Evolutionary conservation profiling

We compared the amino-acid sequences of natural mouse CTRB1 homologs to assess the degree of positional residue conservation due to natural evolution in the substrate binding pocket. A similar analysis was performed for the chymotryptic cleavage-site regions of mouse anionic (T8) and cationic (T7) trypsinogens. The in silico profiling was performed with the ConSurf online tool^22^, as previously described^23^. The required minimal sequence identity with the query sequence was 50%. Using the HMMER algorithm, ConSurf found in the UNIREF90 database 1297 homologues for mouse CTRB1, 1957 for anionic (T8) trypsinogen, and 1915 for cationic (T7) trypsinogen. After exclusion of redundant, highly similar sequences using the CD-HIT algorithm, 150 homologues were sampled for each protein in a balanced way to ensure equal representation with different E-values. Sequences with an incomplete catalytic triad were removed, resulting in 128 mouse CTRB1, 127 anionic (T8) trypsinogen, and 131 cationic (T7) trypsinogen homologues eligible for multiple sequence alignment.

### Structural modeling

The potential structure of the enzyme–substrate complex between the mouse CTRB1 G236R mutant and anionic (T8) trypsinogen was modeled using AlphaFold 3^24^. Predicted interactions between mouse CTRB1 and the Phe150 cleavage site region of mouse anionic (T8) trypsinogen were visualized using PyMol Molecular Graphics Software version 2.6.1.

## Results

### Engineering mouse CTRB1 for higher activity

The mouse CTRB1 (UniProtKB Q9CR35) shares 86% sequence identity with human CTRB1 and CTRB2. It is a functional ortholog of human CTRB2 and bovine chymotrypsin B^15^, as it contains Ala244 rather than Gly244, which results in a slightly narrower substrate-binding S1 pocket. Alignment of the three peptide segments that form the wall of the S1 pocket in mammalian chymotrypsins^25^ indicates a high degree of conservation (Fig. 1). Position 236 is typically occupied by Gly or Ser, except for the Arg and Asp in the human isoforms, while position 244 contains Gly or Ala. The Phe237 residue is unique in mouse CTRB1, as homologues carry a polar residue (Thr, Asn) at this position. In an attempt to convert mouse CTRB1 to a higher activity enzyme, we created single mutants G236R and A244G as well as double mutant G236R-A244G. The wild-type and mutant mouse proenzymes were expressed in HEK 293 T cells and purified from the conditioned medium. After activation with trypsin, the concentration of the chymotrypsin preparations was determined by active-site titration with ecotin.

### Cleavage of chromogenic peptide substrates

First, we determined Michaelis–Menten kinetic parameters on the Suc-Ala-Ala-Pro-Phe-*p*-nitroanilide substrate (Table 1). Compared to wild-type mouse CTRB1, mutants G236R, A244G and G236R-A244G exhibited relatively small changes in the *k*_cat_ and K_M_ values. Thus, *k*_cat_ values were reduced by 1.5-, 1.8- and 1.6-fold, while *K*_M_ values were 3.3-fold lower, 2.4-fold higher, and 1.2-fold lower, respectively. As a result, the catalytic efficiency (*k*_cat_/*K*_M_) of mutant G236R was 2.3-fold increased relative to wild-type mouse CTRB1, whereas those of mutants A244G and G236R-A244G were 4.1-fold and 1.3-fold decreased, respectively. To evaluate the significance of the negatively charged succinyl group at the N terminus of the peptide substrate, we measured enzyme kinetics on the H-Ala-Ala-Pro-Phe-*p*-nitroanilide substrate. Mutants A244G and G236R-A244G did not cleave this peptide to a detectable degree under the conditions tested. In contrast, wild-type mouse CTRB1 exhibited measurable activity with a 1.2-fold lower *k*_cat_ value and a 5.3-fold higher *K*_M_ value, when compared to the succinylated peptide. Mutation G236R decreased the *k*_cat_ by almost twofold with essentially no change in *K*_M_, which resulted in a twofold decrease in catalytic efficiency relative to the wild-type mouse CTRB1. The observations indicate that the mutations caused only modest changes in the activity of mouse CTRB1 on small peptide substrates. Mutation G236R slightly increased catalytic efficiency, which was dependent on the negatively charged N-terminal succinyl group of the peptide. In contrast, mutation A244G reduced catalytic efficiency on the Suc-Ala-Ala-Pro-Phe-*p*-nitroanilide substrate and rendered the enzyme inactive against the H-Ala-Ala-Pro-Phe-*p*-nitroanilide peptide.

**Table 1:** Enzyme kinetic parameters of wild-type and mutant mouse CTRB1 on the Suc-Ala-Ala-Pro-Phe-*p*-nitroanilide and H-Ala-Ala-Pro-Phe-*p*-nitroanilide substrates.

	*K*_M_ (µM)	*k*_cat_ (s^−1^)	*k*_cat_/*K*_M_ (M^−1^∙s^−1^)
Suc-Ala-Ala-Pro-Phe-*p*-nitroanilide			
Mouse CTRB1	30.8 ± 4.4	38.4 ± 2.0	1.2 × 10^6^
G236R	9.3 ± 0.8	26.1 ± 0.6	2.8 × 10^6^
A244G	74.1 ± 10	21.4 ± 1.4	2.9 × 10^5^
G236R-A244G	26.5 ± 4.0	24.1 ± 1.3	9.1 × 10^5^
H-Ala-Ala-Pro-Phe-*p*-nitroanilide			
Mouse CTRB1	162.7 ± 11	31.1 ± 0.9	1.9 × 10^5^
G236R	178 ± 35	16.0 ± 1.3	9.0 × 10^4^

See “Materials & methods” for experimental details. The error of the hyperbolic fits is indicated.

### Digestion of casein

**Fig. 2:**
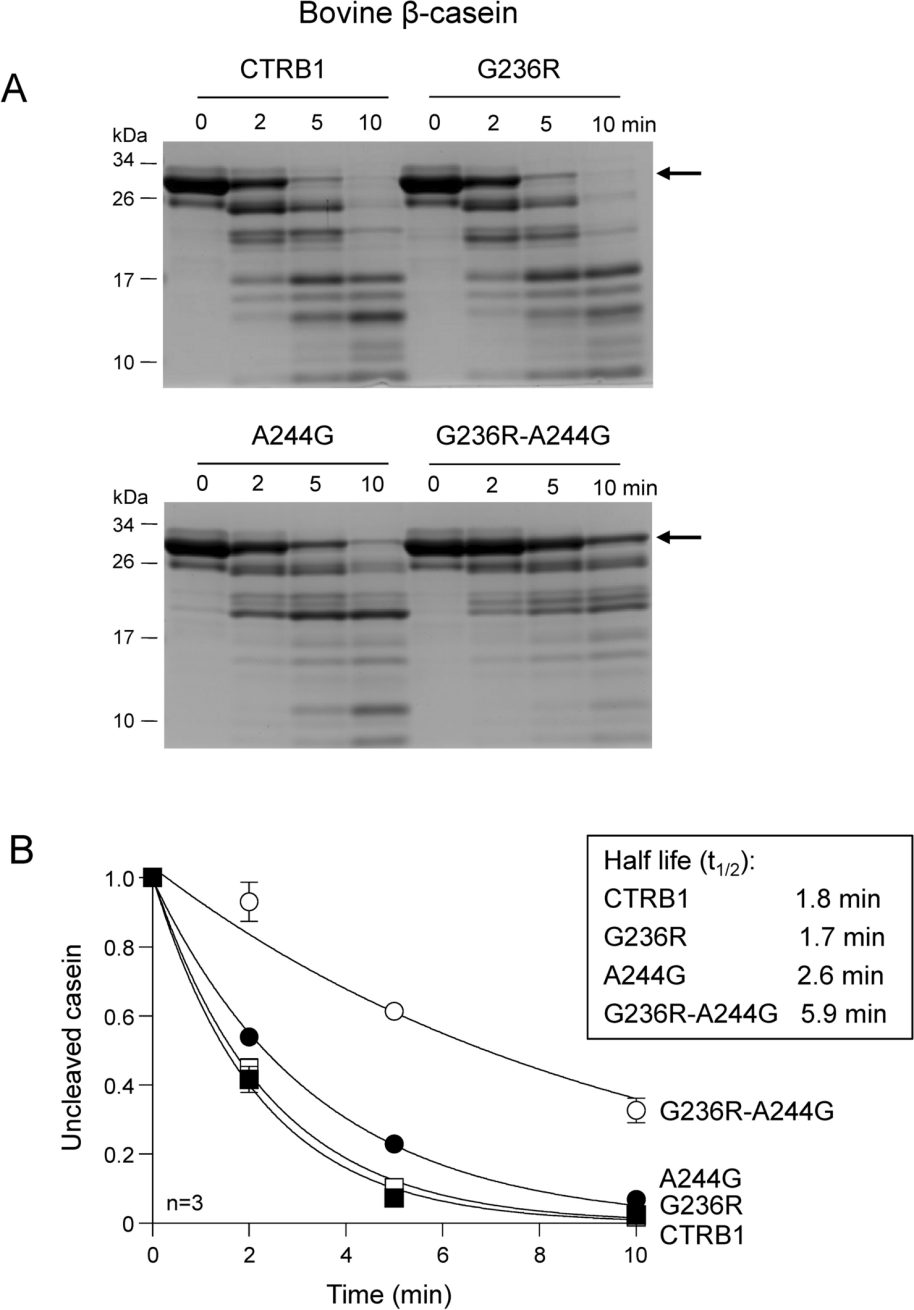
Digestion of bovine β-casein by wild-type mouse CTRB1, single mutants G236R and A244G, and double mutant G236R-A244G. (**A**) Reactions were analyzed by SDS-PAGE and Coomassie Blue staining. Representative gels are shown. (**B**) Casein degradation was quantitated by densitometry. The relative amount of uncleaved casein was plotted as a function of time. The half-life values were calculated from semi-log plots. Data points represent the mean ± SD (n = 3). See “Materials & methods” for experimental details. The arrow indicates the intact, uncleaved protein.

To test chymotrypsin activity on a protein substrate where prime side contacts also contribute to cleavage efficiency, we digested bovine β-casein. All four chymotrypsin constructs readily hydrolyzed casein, as shown by the SDS-PAGE analysis with Coomassie Blue staining (Fig. 2A). Surprisingly, mutation G236R had no impact on casein degradation, whereas mutations A244G and G236R-A244G reduced the rate of digestion by 1.4-fold and 3.3-fold, respectively, as judged by densitometric determination of the half-life values (Fig. 2B). The findings replicate the negative effect of mutation A244G on catalytic activity, as seen with the peptide substrates. The inability of mutation G236R to increase casein digestion by mouse CTRB1 is surprising and stands in contrast with the previously reported 3.4-fold stimulatory effect of mutation D236R in human CTRB1^17^. It appears that elimination of the unfavorable Asp residue in human CTRB1 by mutation D236R was responsible for the activity increase rather than the newly introduced Arg side chain.

### Digestion of mouse anionic (T8) and cationic (T7) trypsinogen

**Fig. 3:**
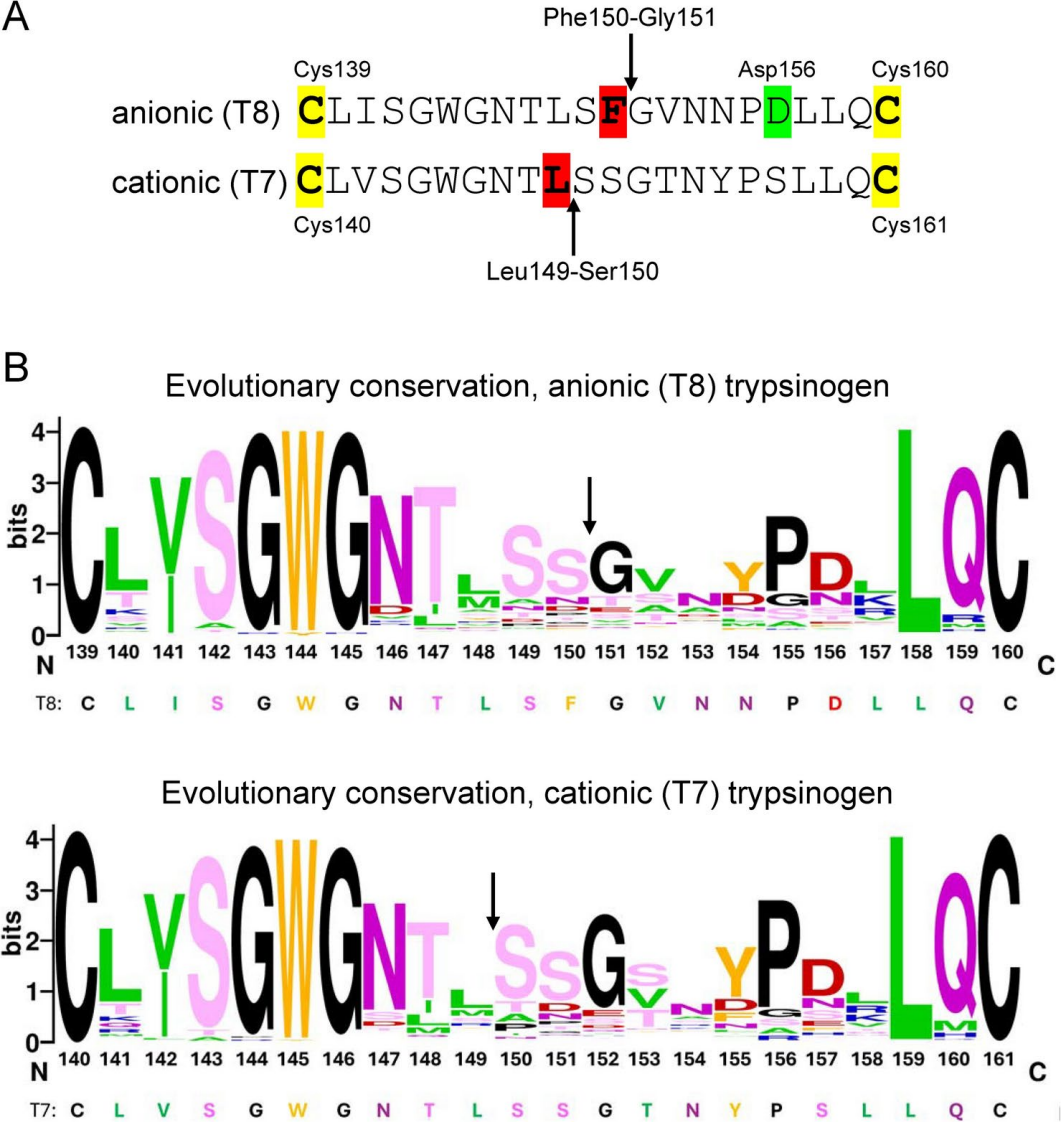
Amino-acid sequence and evolutionary conservation of the peptide segment in mouse anionic (T8) and cationic (T7) trypsinogen cleaved by mouse CTRB1. Note that the amino-acid numbering is shifted by 1 between the two trypsinogen isoforms. (**A**) Chymotrypsin cleavage sites in mouse trypsinogens. The peptide bonds cleaved by mouse CTRB1 are indicated by arrows and the P1 residues are highlighted in red. The conserved Cys residues are shown in yellow, and Asp156 is in green. (**B**) Evolutionary conservation profiling of the chymotryptic cleavage-site regions in mouse anionic (T8) and cationic (T7) trypsinogens. Amino acids are indicated with their one-letter code. The logo was created with the WebLogo application (https://weblogo.berkeley.edu/logo.cgi). The input sequence set was generated by multiple sequence alignment of natural trypsinogen homologs, as described in “Materials & methods”. The incidence of Phe150 and Asp156 among the anionic (T8) trypsinogen homologues were 3.2% and 47.2%, respectively. The height of each column indicates the level of evolutionary conservation (frequency) at the given position. Chemical characteristics of the amino-acid residues are color-coded as follows: aliphatic is green, aromatic is orange, acidic is red, basic is blue, polar without charge is pink, and Cys, Gly, Pro are black. The corresponding amino-acid sequences of mouse T8 and T7 trypsinogens are also shown.

In the context of protection against pancreatitis, the relevant substrate of chymotrypsin is trypsinogen. In the mouse pancreas 85–88% of the trypsinogen content is derived from the cationic T7, and the anionic T8 and T9 isoforms^18^. Since T8 and T9 are 99% identical in their amino-acid sequence, in the present study we used T8 to model the anionic trypsinogens. We previously characterized digestion of mouse anionic (T8) trypsinogen (UniProtKB Q9R0T7) and mouse cationic (T7) trypsinogen (UniProtKB Q9CPN9) with mouse CTRB1 and mouse CTRC^5,6,18^. Anionic (T8) trypsinogen is cleaved at the Phe150-Gly151 peptide bond, whereas the primary cleavage site in cationic (T7) trypsinogen is the Leu149-Ser150 peptide bond (Fig. 3A). Cationic (T7) trypsinogen also suffers secondary cleavages at the Tyr29-Thr30 and Leu159-Gln160 peptide bonds^5^. Evolutionary conservation profiling of the primary cleavage site regions (Fig. 3B) revealed that among homologues of anionic (T8) trypsinogen Phe was rarely found at position 150; where the dominant residue was Ser. In homologues of cationic (T7) trypsinogen, position 149 showed modest conservation with Leu as the most frequently observed residue.

**Fig. 4:**
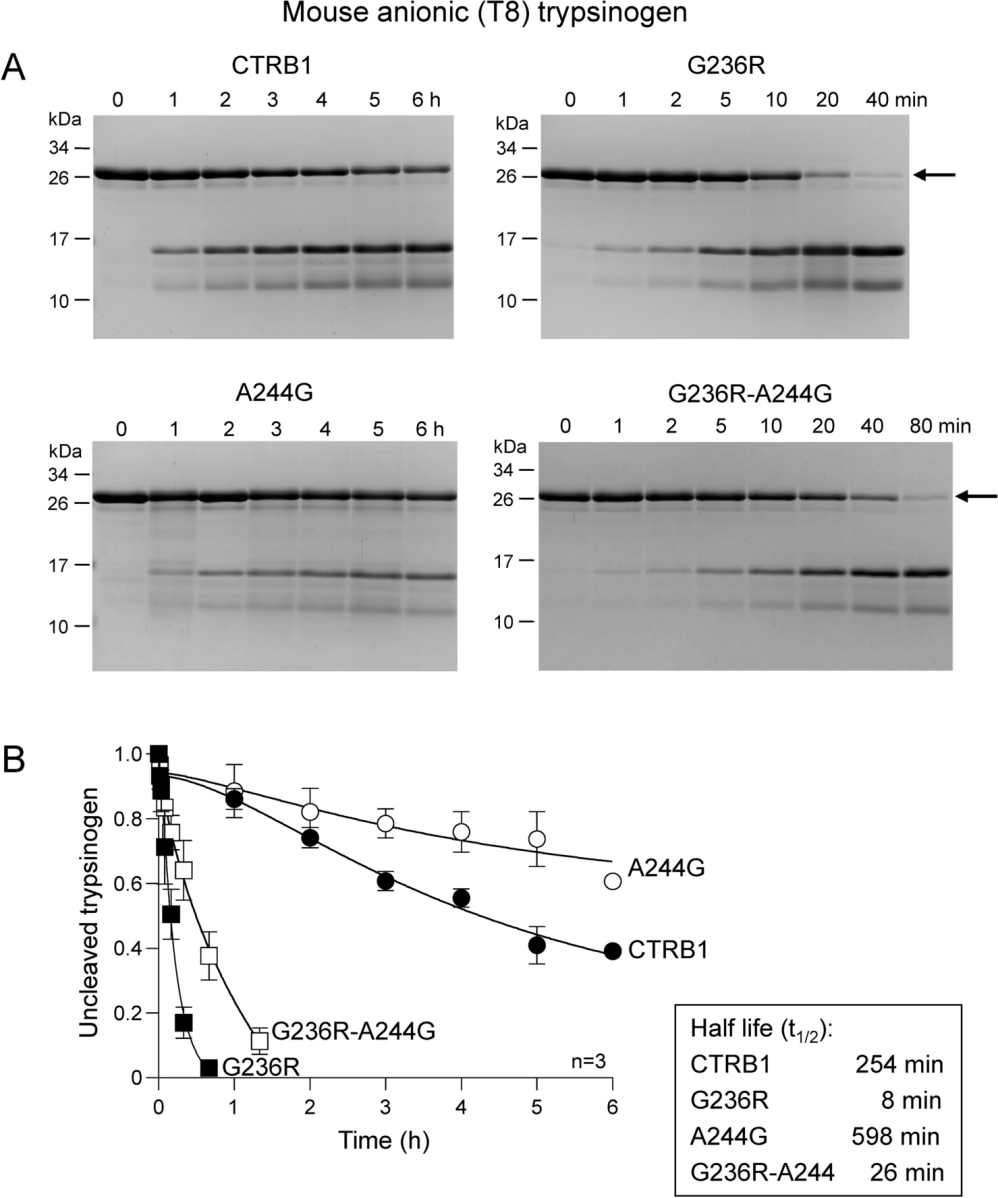
Digestion of mouse anionic (T8) trypsinogen by wild-type mouse CTRB1, single mutants G236R and A244G, and double mutant G236R-A244G. (**A**) Reactions were analyzed by SDS-PAGE and Coomassie Blue staining. Representative gels are shown. (**B**) Trypsinogen cleavage was quantified by densitometry. The relative amount of the uncleaved trypsinogen band was plotted as a function of time. The half-life values were calculated from semi-log plots. Data points represent the mean ± SD (n = 3). See “Materials & methods” for experimental details. The arrow indicates the intact, uncleaved protein.

When purified recombinant mouse anionic (T8) trypsinogen (2 µM) was digested with 1 nM wild-type and mutant mouse CTRB1, a dramatic effect of mutation G236R was shown by the SDS-PAGE pictures (Fig. 4A) and the densitometric evaluation (Fig. 4B, Table 2). Thus, mutant G236R digested T8 trypsinogen 32-fold more efficiently than wild-type CTRB1, as calculated from the measured half-lives. In contrast, mutation A244G reduced the rate of cleavage by 2.4-fold. When the two mutations were combined; the G236R-A244G construct digested anionic (T8) trypsinogen 9.8-fold more rapidly than wild-type CTRB1 but 3.3-fold slower than mutant G236R. Mutation of Phe150 to Ser (F150S) resulted in no detectable digestion with 1 nM mouse CTRB1, and even with 100 nM enzyme only slow cleavage was observed presumably at the Leu148-Ser149 peptide bond (t_1/2_ 307 min) (Table 3). The G236R CTRB1 mutant cleaved the F150S trypsinogen mutant only 1.5-fold better (t_1/2_ 206 min), whereas mutants A244G (t_1/2_ 862 min) and G236R-A244G (t_1/2_ 680 min) showed lower activity than wild-type mouse CTRB1 (Table 3).

**Table 2:** Digestion of mouse anionic (T8) and cationic (T7) trypsinogen by mouse and human chymotrypsins.

Enzyme	Positions 236 and 244	t_1/2_ (min) anionic (T8) trypsinogen 1 nM enzyme	t_1/2_ (min) cationic (T7) trypsinogen 100 nM enzyme
Mouse chymotrypsins			
CTRB1	Gly236, Ala244	254	14
G236R	Arg236, Ala244	8	14
A244G	Gly236, Gly244	598	68
G236R-A244G	Arg236, Gly244	26	79
Human chymotrypsins			
CTRB1	Asp236, Gly244	ND	ND
CTRB2	Arg236, Ala244	10	5
CTRB1 D236R	Arg236, Gly244	11	15

The half-life values (t_1/2_) were determined by densitometric analysis of Coomassie Blue-stained gels. The amino-acid residues that occupy positions 236 and 244 in the chymotrypsin enzymes are indicated. ND, no detectable cleavage.

**Table 3:** Digestion of mouse anionic (T8) trypsinogen mutants F150S and D156S by wild-type and mutant mouse CTRB1.

Enzyme	t_1/2_ (min) F150S 100 nM enzyme	t_1/2_ (min) D156S 1 nM enzyme
CTRB1	307	170
G236R	206	35
A244G	862	ND
G236R-A244G	680	ND

The half-life values (t_1/2_) were measured by densitometric analysis of Coomassie Blue-stained gels. *ND* not determined.

**Fig. 5:**
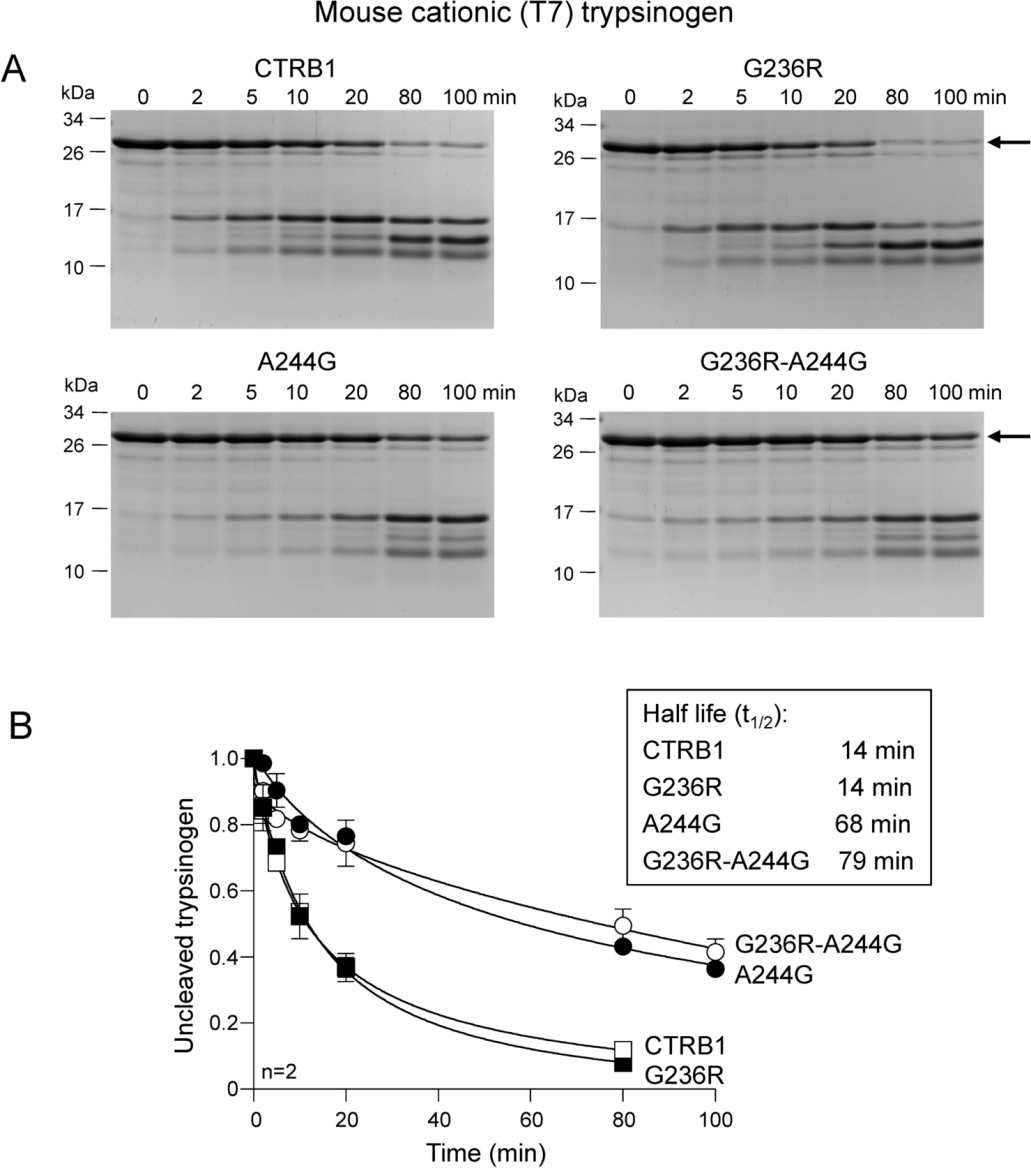
Digestion of mouse cationic (T7) trypsinogen by wild-type mouse CTRB1, single mutants G236R and A244G, and double mutant G236R-A244G. (**A**) Reactions were analyzed by SDS-PAGE and Coomassie Blue staining. Representative gels are shown. (**B**) Trypsinogen cleavage was quantified by densitometry. The relative amount of the intact trypsinogen band was plotted as a function of time. The half-life values were calculated from semi-log plots. Data points represent the mean ± SD (n = 2). See “Materials & methods” for experimental details. The arrow indicates the intact, uncleaved protein.

In contrast to anionic (T8) trypsinogen, the cationic (T7) isoform does not contain the highly sensitive Phe151-Gly152 peptide bond (note that numbering is shifted by 1, see Fig. 3). Consequently, cationic (T7) trypsinogen was digested much slower at the Leu149-Ser150 peptide bond (Fig. 5A); 100 nM mouse CTRB1 hydrolyzed half of this substrate in 14 min (Fig. 5B, Table 2). Remarkably, as seen with the F150S anionic (T8) trypsinogen mutant, no increase in digestion was observed with the G236R CTRB1 mutant (t_1/2_ 14 min). Mutation A244G reduced the rate of cleavage 4.9-fold (t_1/2_ 68 min) and the double mutant G236R-A244G showed similar behavior (t_1/2_ 79 min). Thus, the stimulatory effect of mutation G236R on mouse CTRB1-mediated cleavage appears to be substrate dependent.

### Role of Asp156 in chymotryptic cleavage of mouse anionic (T8) trypsinogen

**Fig. 6:**
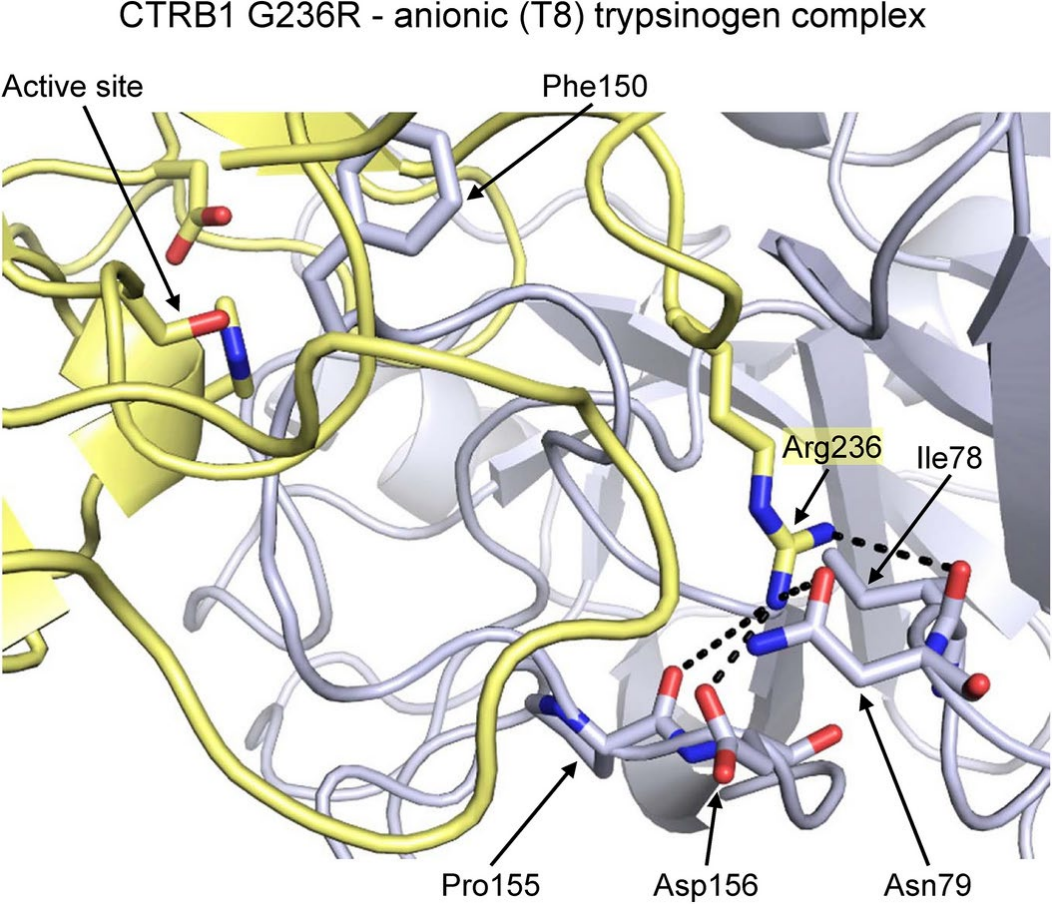
Potential enzyme–substrate interactions between the G236R mouse CTRB1 mutant and mouse anionic (T8) trypsinogen. Magnified view of the interaction between the Phe150 cleavage site region of anionic (T8) trypsinogen (gray) and the substrate binding pocket of mouse CTRB1 (yellow) rendered in cartoon representation. Presumed hydrogen bonds formed by the guanidino group of Arg236 with the indicated side-chain and main chain atoms of anionic (T8) trypsinogen are represented by dashed lines. The “active site” label refers to the catalytic triad of chymotrypsin.

The sequence of the peptide segment between Cys139 and Cys160 in mouse anionic (T8) trypsinogen and the homologous Cys140-Cys161 region in cationic (T7) trypsinogen are highly similar with only a few notable differences (Fig. 3A). Besides the unique Phe150, the anionic (T8) isoform also contains an acidic amino acid, Asp156. We modeled the interaction between mouse CTRB1 mutant G236R and the Phe150 cleavage site region of anionic (T8) trypsinogen using AlphaFold 3 (Fig. 6). A hypothetical complex showed that the side chain of Arg236 could form multiple interactions with anionic (T8) trypsinogen, including H-bonds with the side chains of Asn79 and Asp156, and the main chain atoms of Ile78 and Pro155. Based on this model, we speculated that the Asp156 side chain in anionic (T8) trypsinogen might contribute to the enhanced cleavage seen with the G236R mouse CTRB1 mutant through electrostatic and H-bonding interactions with Arg236. To test this notion, we generated the D156S anionic (T8) trypsinogen mutant and measured rates of digestion with wild-type mouse CTRB1 and mutant G236R. The results (Table 3) indicated that 1 nM mutant G236R digested the D156S mutant trypsinogen only 4.9-fold faster (t_1/2_ 35 min) than wild-type mouse CTRB1 (t_1/2_ 170 min), as compared to the 32-fold difference seen on wild-type anionic (T8) trypsinogen. The observations suggest that Asp159 in anionic (T8) trypsinogen is an important determinant of cleavage efficiency by the G236R CTRB1 mutant.

### Digestion of mouse trypsinogens with human CTRB1, CTRB2 and CTRB1 D236R mutant

The variable effect of mutation G236R on the cleavage of various peptide and protein substrates by mouse CTRB1 was puzzling because in our previous work introduction of Arg236 into human CTRB1 resulted in an improved enzyme on all substrates tested^17^. To investigate this phenomenon further, we digested mouse anionic (T8) and cationic (T7) trypsinogen with human CTRB1, CTRB2 and the CTRB1 D236R mutant. Using 1 nM enzyme, we found that anionic (T8) trypsinogen was not digested by human CTRB1 to a detectable degree whereas it was rapidly cleaved by human CTRB2 and the CTRB1 D236R mutant at comparable rates (t_1/2_ 10 min and 11 min, respectively, Table 2). Similarly, using 100 nM enzyme concentration, human CTRB1 was unable to degrade cationic (T7) trypsinogen while human CTRB2 and the CTRB1 D236R mutant digested this isoform efficiently (t_1/2_ 5 min and 15 min, respectively, Table 2). Interestingly, human CTRB2 and the CTRB1 D236R mutant had comparable activity on anionic (T8) trypsinogen but CTRB2 was nearly threefold better on cationic (T7) trypsinogen. Previously, we reported that the human CTRB1 D236R mutant degraded human anionic trypsinogen 7.1-fold more rapidly than CTRB2^17^. Taken together, the observations indicate that the stimulatory effect of the Arg236 side chain is strongly context and substrate dependent even when tested on highly homologous trypsinogen paralogs as substrates.

## Discussion

In our recent publication, we demonstrated that human CTRB2 was a better chymotrypsin than human CTRB1 while the efficacy of bovine CTRA lay between those of the human isoforms^17^. Furthermore, introducing the Arg236 side chain into human CTRB1 by mutation D236R converted this isoform to a CTRB2-like high activity protease. The observations identified Arg236 in human CTRB2 as a positive determinant, while Asp236 in human CTRB1 as a negative determinant for chymotrypsin activity and provided a plausible biochemical mechanism for the protective effect of the *CTRB1-CTRB2* inversion allele against chronic pancreatitis^17^.

Inspired by these earlier findings, in the present study we set out to engineer mouse CTRB1 for improved chymotrypsin activity, with the ultimate goal of generating an enzyme capable of rapidly degrading mouse trypsinogens and thereby possessing an enhanced ability to protect against pancreatitis in mouse models. One of the surprising results of our previous study was that the human CTRB1 D236R mutant exhibited superior degrading activity on human anionic trypsinogen, even 7.1-fold better than the human CTRB2 isoform^17^. This suggested to us that the presence of Gly244 in human CTRB1 (versus Ala244 in human CTRB2) further improved trypsinogen degradation when present together with Arg236. Therefore, in our experimental design we replaced not only Gly236 with Arg (mutant G236R) in the mouse CTRB1 but also changed Ala244 to Gly, alone (mutant A244G) or in combination with the G236R mutation (double mutant G236R-A244G). The protease activity of the wild-type and three mutant mouse CTRB1 enzymes were tested on peptide substrates, bovine β-casein, and mouse anionic (T8) and cationic (T7) trypsinogens.

The results showed that in the context of mouse CTRB1, these mutations had substrate-specific effects that were different from those observed with the human CTRB1 D236R mutant. On chromogenic peptide substrates, relatively small differences were observed. Mutation G236R slightly increased catalytic efficiency of mouse CTRB1, which was dependent on the negatively charged N-terminal succinyl group of the peptide, whereas mutation A244G reduced activity. In casein digestion experiments, mutation G236R had no stimulatory effect, while mutations A244G and G236R-A244G somewhat decreased the rate of digestion by mouse CTRB1. In contrast, previously we found that the D236R mutation in human CTRB1 increased catalytic efficiency on the Suc-Ala-Ala-Pro-Phe-*p*-nitroanilide peptide substrate by 12.5-fold, and increased casein digestion by 3.4-fold, relative to wild-type human CTRB1^17^. The comparison of the human and mouse data suggest that on the peptide and casein substrates the beneficial effect of the human D236R mutation may have derived from the removal of the Asp side chain rather than the introduction of Arg.

The substrate-dependence of the stimulatory effect by Arg236 was also apparent in the trypsinogen digestion experiments, where mutation G236R markedly enhanced cleavage of anionic (T8) trypsinogen by mouse CTRB1 but had no effect on the degradation of cationic (T7) trypsinogen. Mutation A244G had a negative effect on the digestion of both trypsinogen isoforms. Previously, we found that human CTRB2 (carrying Arg236) and the human CTRB1 D236R mutant (also carrying Arg236) degraded human anionic trypsinogen 5.3-fold and 37.7-fold more efficiently, respectively, than wild-type human CTRB1^17^. The 7.1-fold enhanced activity of the human D236R CTRB1 mutant relative to human CTRB2 was unexplained since both enzymes contained Arg236 and suggested that somehow the functional interaction of Arg236 and Gly244 (present in CTRB1 but absent in CTRB2) was responsible for the phenomenon. However, in the present experiments, Gly244 consistently reduced chymotrypsin activity on all substrates, even if paired with Arg236. Interestingly, when the human enzymes were tested against the two mouse trypsinogen isoforms, the stimulatory effect of Arg236 was evident with both substrates. Thus, CTRB1 poorly digested mouse anionic (T8) and cationic (T7) trypsinogens but human CTRB2 and the CTRB1 D236R mutant rapidly cleaved both isoforms. In contrast to previous observations with the human anionic trypsinogen substrate, here the human CTRB1 D236R mutant was not superior to human CTRB2 in its trypsinogen-degrading activity; anionic (T8) trypsinogen was cleaved at similar rates by the two enzymes and cationic (T7) trypsinogen was digested threefold faster by human CTRB2.

The observations are best interpreted with the help of structural modeling. As reported previously^17^, due to the presence of Asp236 in human CTRB1, the Met210 side chain partially obstructs the substrate binding groove, which is relieved by the D236R mutation. This obstruction was not apparent in bovine CTRA, which contains a neutral Ser residue at position 236. The bovine enzyme exhibited intermediate activity on the various substrates tested, relative to human CTRB1 and CTRB2. It is likely that mouse CTRB1, which harbors the neutral Gly residue at position 236, adopts a similar conformation as the bovine enzyme with respect to the position of Met210, and the accessibility of the substrate binding site. Consequently, mutation G236R in mouse CTRB1 does not recapitulate the full effect of mutation D236R in human CTRB1, inasmuch as it does not alleviate an inhibitory configuration. Rather, the strong positive effect of mutation G236R on the digestion of anionic (T8) trypsinogen by mouse CTRB1 may be related to the unique features of the peptide segment cleaved, such as the Phe150 cleavage site, which is rarely found in homologues, as shown by evolutionary conservation profiling. The Arg236 side chain in the G236R CTRB1 mutant might form a H-bridge with Asp156 of mouse anionic (T8) trypsinogen and a direct electrostatic attraction between Arg236 and Asp156 is also likely. We found that the D156S mutation in anionic (T8) trypsinogen significantly reduced the stimulatory effect of Arg236 on cleavage at Phe150, confirming that Asp156 is an important determinant of accelerated substrate cleavage by mouse CTRB1.

The results from this study are expected to inform the design of preclinical mouse models with higher trypsinogen degradation ability and enhanced resilience against pancreatitis. Increasing protective chymotrypsin activity inside the pancreas is a therapeutic option against chronic pancreatitis and a mouse model can offer proof of concept for this approach. In the clinical setting, chymotrypsin-based therapy might be administered via intraductal injection of recombinant adeno-associated virus (AAV) vectors. A recent study in mice demonstrated that an AAV8-based vector carrying the trypsin inhibitor SPINK1 could be successfully targeted to the pancreas resulting in significant protection against pancreatitis^26^. These findings pave the way for the testing of AAV-mediated intrapancreatic delivery of chymotrypsin for the treatment of the human disease.

## Supplementary Information

Supplementary Information.

## Data Availability

Experimental data are provided within the manuscript.
