# Supplementary material for: Engineering mouse chymotrypsin B1 for improved trypsinogen degradation
Source: Sci Rep. 2025 Mar 25;15:10201. doi: 10.1038/s41598-025-94299-1 (PMC11937528; doi:10.1038/s41598-025-94299-1)

Uncropped gel pictures for Figure 2A

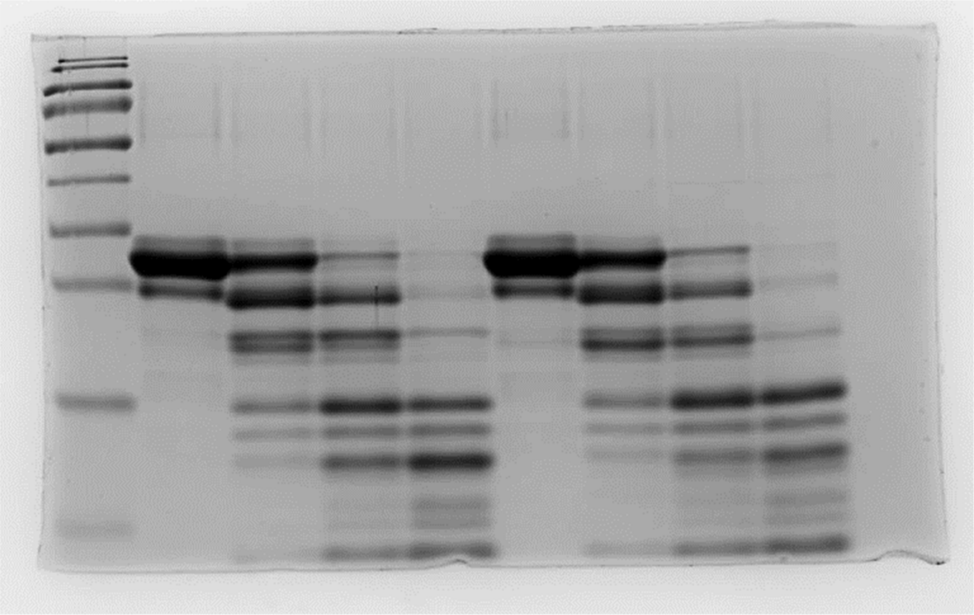

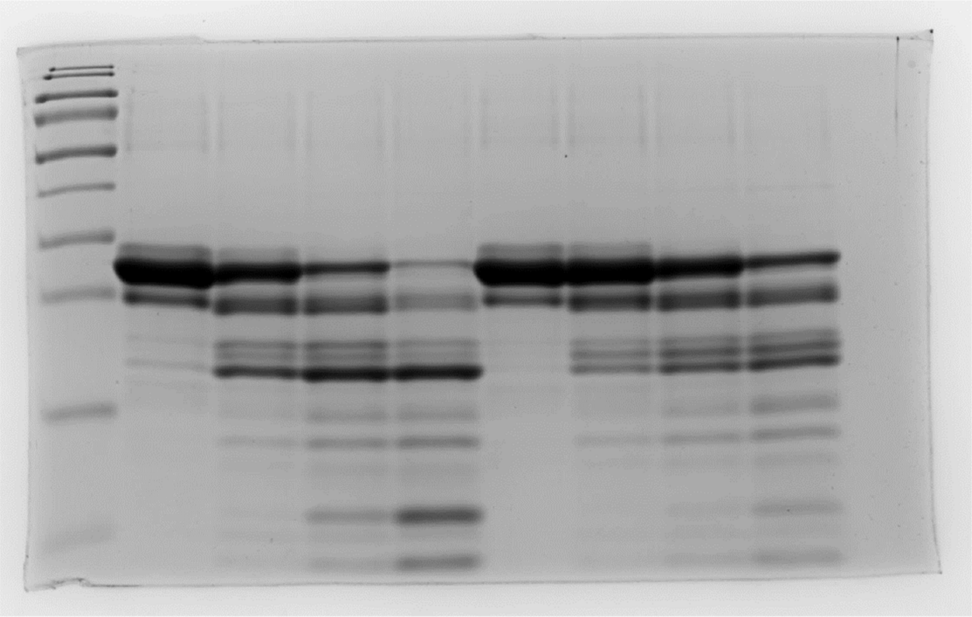

Uncropped gel pictures for Figure 4A

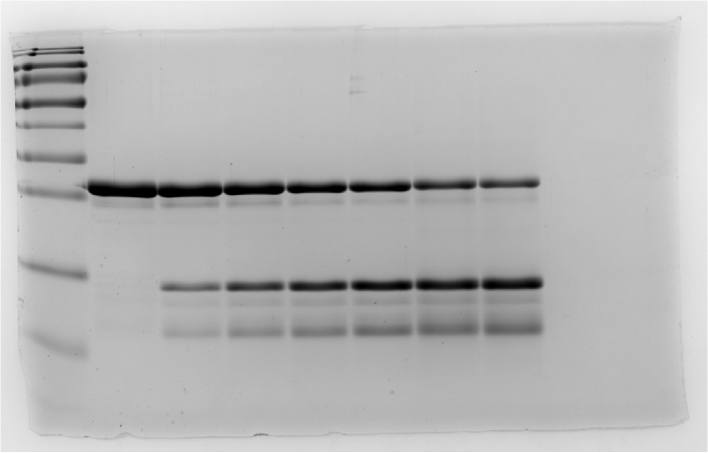

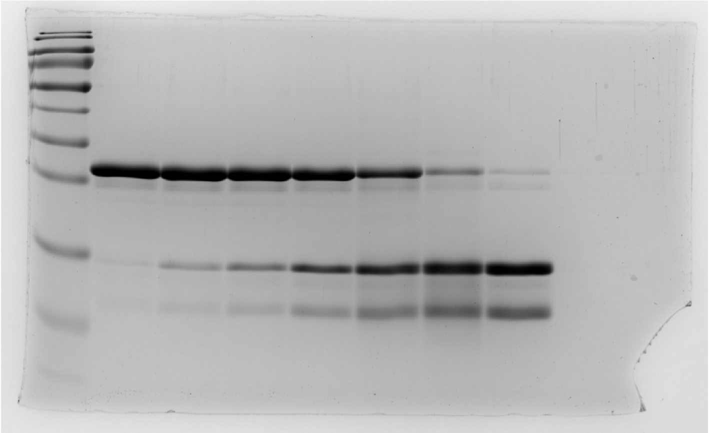

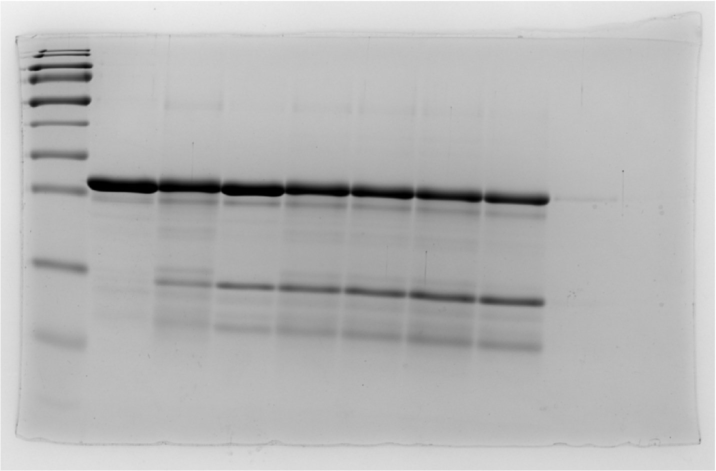

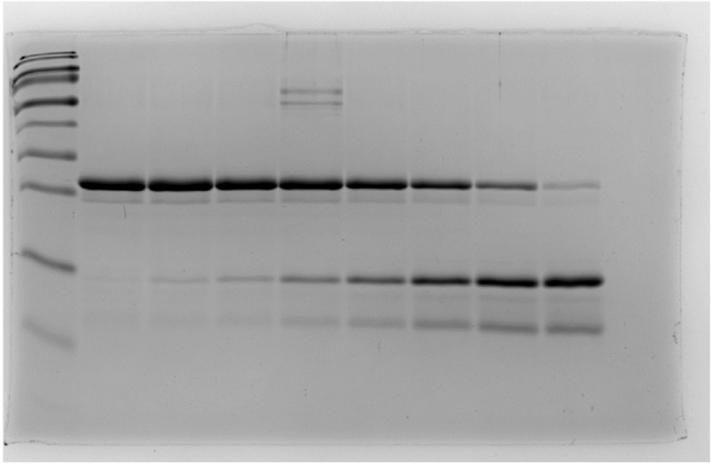

Uncropped gel pictures for Figure 5A

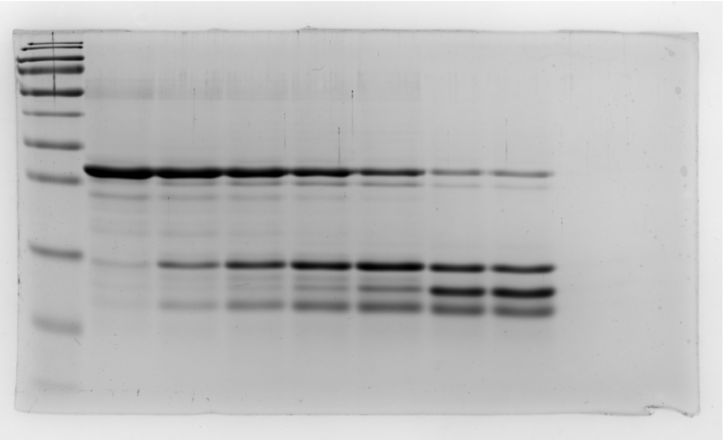

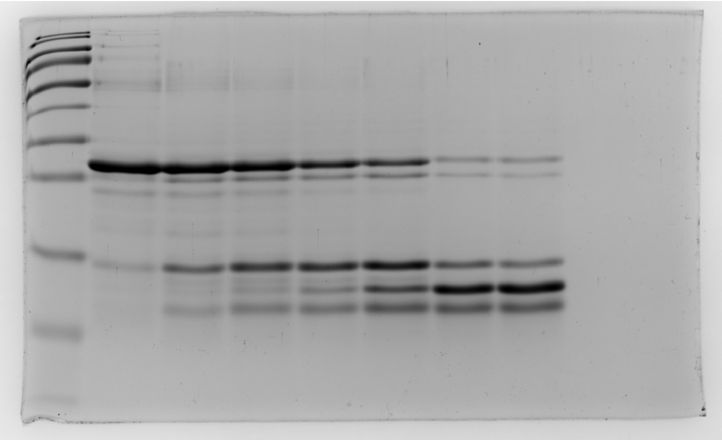

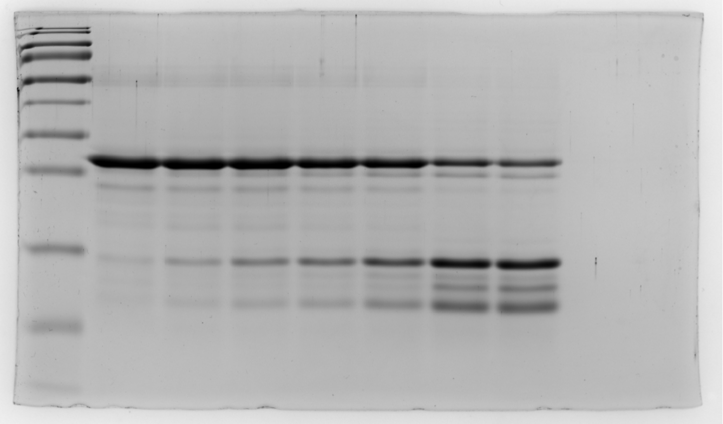

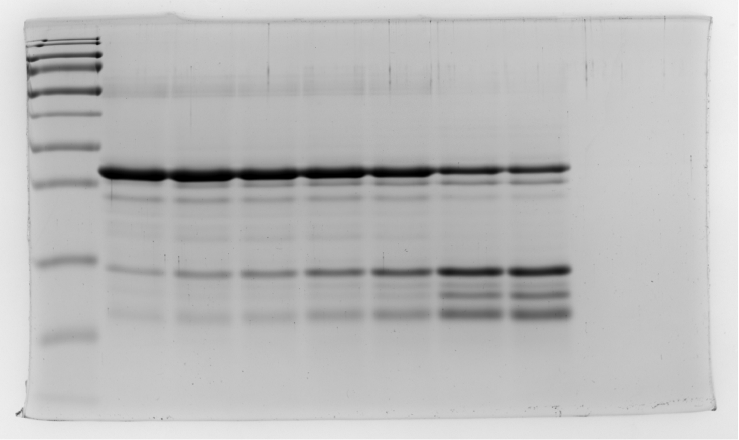

Supplement: Supplementary file 1 — Supplementary Information. [file 41598_2025_94299_MOESM1_ESM.pdf]
